# Prevalence of Swallowing and Eating Difficulties in an Elderly Postoperative Hip Fracture Population—A Multi-Center-Based Pilot Study

**DOI:** 10.3390/geriatrics5030052

**Published:** 2020-09-16

**Authors:** Gitte Madsen, Stine M. Kristoffersen, Mark R. Westergaard, Vivi Gjødvad, Merete M. Jessen, Dorte Melgaard

**Affiliations:** 1 Department of Physiotherapy and Occupational Therapy, Randers Regional Hospital, Skovlyvej 15, 8930 Randers, Denmark; gittmads@rm.dk (G.M.); vivirasm@rm.dk (V.G.); merejess@rm.dk (M.M.J.); 2 Department of Physiotherapy and Occupational Therapy, Horsens Regional Hospital, Sundvej 30, 8700 Horsens, Denmark; stimoa@rm.dk; 3 Department of Physiotherapy and Occupational Therapy, North Denmark Regional Hospital, Bispensgade 37, 9800 Hjoerring, Denmark; mark.westergaard@rn.dk; 4 Department of Clinical Medicine and Center for Clinical Research, Aalborg University and North Denmark Regional Hospital, Bispensgade 37, 9800 Hjoerring, Denmark

**Keywords:** swallowing difficulties, eating difficulties, dysphagia, swallowing disorder, hip fracture, orthopedic surgery, elderly

## Abstract

Elderly patients operated for hip fracture are characterized by high age and high degree of comorbidity and need of care, factors previously found to be associated with swallowing and eating difficulties. The aim of this study was to investigate the prevalence of swallowing and eating difficulties in an elderly postoperative hip fracture population and to identify factors associated with swallowing and eating difficulties. A cross-sectional multi-center pilot study was performed, including patients ≥65 years, operated for hip fracture, and able to participate in a swallowing and eating assessment. A clinical assessment was conducted using Danish versions of the standardized tools Volume-Viscosity Swallow Test and Minimal Eating Observation Form-version II. Demographic data and clinical characteristics were examined. A total of 78 patients (mean age 81.4 years (SD 7.8), 30.8% male) were included. Swallowing and eating difficulties were present in 60 patients (77%). Swallowing and eating difficulties were significantly associated with living in a nursing home before hospital admission (*p* = 0.014), low habitual New Mobility Score (*p* = 0.018), and absence of cardiac comorbidity (*p* = 0.023). The results underline the importance of focusing on swallowing and eating difficulties in elderly patients operated for hip fracture to ensure effectivity and safety and optimize the prognosis for the patient.

## 1. Introduction

According to the International Classification of Functioning, Disability and Health (ICF), swallowing is classified as “functions of clearing substances, such as food, drink or saliva through the oral cavity, pharynx and esophagus into the stomach at an appropriate rate and speed” (b5105) and eating as “carrying out the coordinated tasks and actions of eating food that has been served, bringing it to the mouth and consuming it in culturally acceptable ways, cutting or breaking food into pieces, opening bottles and cans, using eating implements, having meals, feasting or dining” (d550) [1]. Swallowing and eating difficulties, the focus of this study, describe challenges in meeting these basic needs. Swallowing and eating difficulties include dysphagia, which is defined as a geriatric syndrome [2]. However, the ability to swallow and eat efficiently and securely depends not only on the presence or absence of dysphagia. Other prerequisites are the ability to adopt and maintain a good sitting position, the ability to handle the food on the plate and transport it to the mouth, the ability to manipulate the food in the mouth, and having enough energy to eat a complete meal [3,4].

Dysphagia and eating difficulties have been reported to be highly prevalent among the elderly [2,4,5,6,7,8,9,10,11]. As a result of the normal aging process, anatomical and physiological changes occur to the swallowing- and eating-related structures, such as muscular weakness in the throat, osteoporotic fractures in the neck, and sensibility disturbances [11,12,13,14,15,16,17,18]. The consequences of dysphagia are malnutrition, dehydration, aspiration, pneumonia, frailty, reduced quality of life, depression, social withdrawal, and mortality [8,11,18,19,20,21,22,23]. Economically, the consequences are also high, given the fact that people with dysphagia are hospitalized and re-hospitalized more often and have an increased hospital length of stay compared to people without these difficulties [8,21,24,25].

Due to the progressive aging of the population, the incidence of hip fracture is continuing to rise worldwide [26]. In the year 2000, the worldwide incidence of hip fracture was estimated to be more than 1.6 million [27], and the global number of hip fractures is expected to rise to 4.5 million by the year 2050 [26]. The risk of dying increases after hip fracture; mortality has been reported to range from 7.5% to 13.3% 30 days/1 month following surgery [28,29,30,31] and from 8.4% to 36% one year after the operation [28,32,33,34]. Furthermore, re-hospitalization within 28–30 days after being discharged has been reported to range from 8.3% to 11.9% [35,36,37]. Early re-hospitalization after hip fracture surgery is often caused by pneumonia, dehydration, and loss of functional capacity [36]. It is previously found that pneumonia due to aspiration, dehydration, reduced functional capacity, increased risk of re-hospitalization, and increased mortality is closely related to dysphagia [2,8,11,21,22].

Patients operated for hip fracture are characterized by several factors previously found to be associated with different kinds of swallowing and eating difficulties, including high age and a high degree of comorbidity and need of care [11,26,38]. Despite this, only a few studies have focused on swallowing and eating difficulties in an elderly postoperative hip fracture population. We found four studies focusing on dysphagia. These studies documented a prevalence of dysphagia of 5.3–54% for patients ≥65 years operated for hip fracture [39,40,41,42]. To our knowledge, no studies have previously focused on swallowing and eating difficulties in a broader perspective in an elderly postoperative hip fracture population.

The primary aim of this study was to investigate the prevalence of swallowing and eating difficulties in an elderly postoperative hip fracture population and secondly to identify factors associated with swallowing and eating difficulties.

## 2. Materials and Methods

### 2.1. Design and Patient Sample

A cross-sectional multi-center pilot study was performed at the Departments of Orthopedic Surgery at North Denmark Regional Hospital (RHN), Randers Regional Hospital (RRA), and Horsens Regional Hospital (RHH) in a six-week period at each hospital from May to November 2019. Patients ≥65 years operated for hip fracture and able to participate in a swallowing and eating assessment were included. Patients fully nourished with probe upon admission and patients who were discharged before a swallowing and eating assessment were excluded. Furthermore, patients with severe dementia or severe cognitive impairment were excluded because they were not able to contribute to the swallowing and eating assessment.

### 2.2. Swallowing and Eating Assessment

A clinical swallowing and eating assessment was conducted by an experienced occupational therapist postoperatively. The Danish versions of the standardized tools Volume-Viscosity Swallow Test (V-VST) [43,44,45] and Minimal Eating Observation Form-version II (MEOF-II) [3,4,46] were used.

In V-VST, three different viscosities are used in three different volumes (5, 10, and 20 mL). The bolus viscosity was liquid viscosity (21.61 mPa.s), nectar viscosity (295.02 mPa.s) achieved by adding 1.2 g of the thickener Resource ThickenUp (Nestlé HealthCare Nutrition) to 100 mL water, and pudding viscosity (3682.21 mPa.s) achieved by adding 6.0 g of the thickener Resource ThickenUp to 100 mL water. Water at room temperature was used. Boluses of each volume and viscosity were offered to the patient with a disposable syringe. Oxygen saturation was measured before and during the test using a pulse oximeter on the patient’s index finger. V-VST assesses dysfunction in swallowing regarding effectivity and safety. According to the test, signs of impaired effectivity are impaired labial seal, oral or pharyngeal residue, and/or incomplete sinking. Signs of impaired safety are changes of voice quality, cough, and/or decrease in oxygen saturation ≥3% to detect silent aspiration. One or more signs of impaired effectivity or safety indicate swallowing difficulties [43,44,45].

MEOF-II is a systematic observation of a meal with a variation of viscosities. The test includes observations related to three categories of eating-related disabilities: (1) ingestion, (2) deglutition, and (3) energy/appetite. Each category contains three sub-questions: (1) ingestion includes “sitting position,” “manipulation of food on the plate,” and “transport of food to the mouth,” (2) deglutition includes “manipulation of food in the mouth,” “swallowing,” and “ability to chew,” and (3) energy/appetite includes “eating less than 3/4 of served food,” “energy to eat until having satisfied hunger,” and “appetite compared to previously” [3,4,46,47].

In this study, eating difficulties assessed through the MEOF-II were categorized into no eating difficulties or eating difficulties. Patients with a dysfunction in ingestion, deglutition, and/or energy/appetite regarding the sub-question “energy to eat until having satisfied hunger” were considered to have swallowing and eating difficulties. Patients with a dysfunction only in energy/appetite regarding the sub-questions “eating less than ¾ of served food” and/or “appetite compared to previously” were not considered to have swallowing and eating difficulties since it is well known that many patients experience nausea and decreased appetite after surgery [48,49].

If either the V-VST or MEOF-II test was positive, the patient was considered to have swallowing and eating difficulties.

### 2.3. Other Variables

Demographic data were gender, age, body mass index (BMI), and habitual housing form and clinical factors score according to the American Society of Anesthesiologists (ASA score), comorbidity, fracture type, time from admission to surgery, anesthesia type, surgery type, time from surgery to swallowing and eating assessment, presence of delirium according to The Confusion Assessment Method (CAM) and Cumulated Ambulation Score (CAS score) day 1 after surgery. These data were obtained from medical records. Habitual New Mobility Score (NMS) and knowledge of swallowing difficulties demonstrated before the hip fracture were obtained based on self-reporting from the patient, a relative, or a care assistant.

### 2.4. Data Analysis

Study data were collected and managed using Research Electronic Data Capture tool (REDCap) hosted at North Denmark Region. REDCap is a secure, web-based software platform designed to support data capture for research studies [50,51].

Descriptive statistics were used to summarize the demographic, preoperative, intraoperative, and postoperative characteristics of the population and to document the prevalence of swallowing and eating difficulties. Categorical data were reported by number (n) and percent (%) (Fisher’s exact test) and continuous data by mean and standard deviation (SD) (t-test). Continuous data that did not meet the assumption of normal distribution were reported by median and interquartile range (IQR).

All statistical analyses were performed using Stata version 13.1 (Stata Corporation, College Station, Texas, TX, USA). A *p*-value ˂ 0.05 was considered statistically significant.

### 2.5. Ethics

Assessment of swallowing and eating difficulties is common practice in Denmark, but not systematically. This is a quality development project where the assessment was performed systematically, and therefore, the regional ethical committee of Northern Denmark waived the need for approval. The study was registered with the Danish Data Protection Authority (2008-58-0028).

## 3. Results

**Figure 1:**
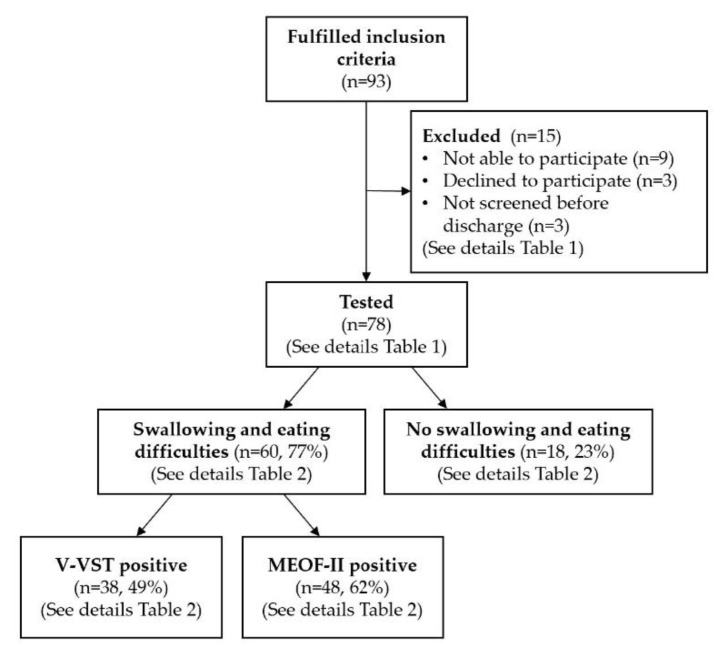
Flowchart describing exclusion of participants and prevalence of swallowing and eating difficulties.

As presented in Figure 1, 93 patients were operated for hip fracture during the time of inclusion, 15 patients were excluded, and 78 patients (84%) were tested with V-VST and MEOF-II for swallowing and eating difficulties (RRA: *n* = 45, RHN: *n* = 17, RHH: *n* = 16). Out of 78 patients, 60 patients tested positive, ending up with a prevalence of swallowing and eating difficulties of 77%.

As illustrated in Table 1, patients screened had a mean age of 81.4 (SD 7.8) and 30.8% were male.

A larger proportion of patients screened lived habitually in their own residence (*p* = 0.048). Patients screened waited on average shorter time from admission to surgery (*p* = 0.030) and fewer underwent surgery in general anesthesia (*p* = 0.006). Patients screened were less likely to be delirious (*p* = 0.024), and they had a higher CAS-score day 1 after surgery (0.003).

As presented in Table 2, of the 60 patients tested positive for swallowing and eating difficulties, 17 patients (28.3%) showed impaired safety and 32 (53.3%) impaired efficacy using the V-VST. Altogether, 38 patients (63.3% of patients tested positive) had a positive V-VST. Of the 60 patients tested positive for swallowing and eating difficulties, 48 patients (80%) showed a dysfunction in ingestion, 38 patients (63.3%) in deglutition, and 19 patients (31.7%) in energy/appetite using the MEOF-II. Thirty-eight patients (63.3%) showed a dysfunction in the ability to adopt and maintain a good sitting position. Altogether, 48 patients (80.0% of patients tested positive) had a positive MEOF-II.

A larger proportion of patients with swallowing and eating difficulties lived habitually in nursing homes (*p* = 0.014), and fewer had cardiac comorbidity (*p* = 0.023). Mean habitual NMS was lower for patients with swallowing and eating difficulties (*p* = 0.018).

## 4. Discussion

This study documented a prevalence of 77% of swallowing and eating difficulties in an elderly postoperative hip fracture population. Living in a nursing home before hospital admission, a low habitual NMS, and the absence of cardiac comorbidity were found to be significantly associated with swallowing and eating difficulties.

Previous studies have focused on the prevalence of dysphagia following hip fracture surgery and documented a prevalence of 5–54% for patients ≥65 years operated on for hip fracture [39,40,41,42]. Due to the broader perspective on swallowing and eating difficulties in this study combined with different ways of assessing the difficulties, a direct comparison of results is impossible. However, the V-VST test results can be compared to previous studies. This study documented dysfunction in swallowing regarding effectivity and/or safety assessed by the V-VST in 49% of the patients tested. This is higher than the prevalence of dysphagia documented by Love et al. (2013), who found a prevalence of 34% [41], but comparable to prevalence of 42% and 54% documented by Meals et al. (2016) and Beric et al. (2019), respectively [39,42]. Byun et al. (2019) found a prevalence of dysphagia of 5% [40]. However, results are not comparable because only patients who were considered at high risk of dysphagia, based on patient history, patient-reported symptoms of dysphagia, and a simple water swallowing test, underwent an assessment in that study.

In this study, living in a nursing home before hospital admission was found to be significantly associated with swallowing and eating difficulties. This supports the findings in previous studies focusing on patients with hip fracture [39,41] and on elderly patients in general [8]. In Denmark, due to the characterization of patients with hip fracture including high age and a high degree of comorbidity, patients living in a nursing home before hospital admission are often discharged from the hospital very shortly after the operation to avoid delirium. There is only a very short time postoperatively in the hospital to focus on swallowing and eating difficulties, and often the patient is discharged before a swallowing and eating assessment is performed. The results in the present study and the previous studies mentioned highlight the importance of caregivers focusing on possible swallowing and eating difficulties in patients living in a nursing home.

Furthermore, a low habitual NMS was found to be significantly associated with swallowing and eating difficulties in this study. NMS is a validated predictor of long-term mortality and rehabilitation outcome in patients with hip fracture [52]. The finding of an association between swallowing and eating difficulties and a low habitual NMS supports previous findings of an association between eating difficulties and reduced activity of daily living and may indicate the risk of long-term mortality and not optimal outcome of rehabilitation [9,52]. Finally, the absence of cardiac comorbidity was found to be significantly associated with swallowing and eating difficulties in this study, which most likely is a random finding caused by the small sample size.

All the patients who showed signs of swallowing and eating difficulties assessed by MEOF-II had problems with ingestion. Particularly, a large proportion of the population showed difficulties in the ability to adopt and maintain a good sitting position during the MEOF-II assessment. Previous studies conclude that sitting position is essential for the ability to swallow and eat efficiently and securely [53,54,55]. The large proportion of patients with a poor sitting position highlights the importance of a caregiver’s postural modification of the patient with hip fracture before meals to optimize the requisites for swallowing and eating efficiently and securely.

### Strengths and Limitations

To our knowledge, no studies have previously focused on swallowing and eating difficulties in a broader perspective in an elderly postoperative hip fracture population. The broader perspective enables a focus on several important prerequisites to swallowing and eating that are highly relevant for the population, for instance their sitting position. Therefore, the broader perspective is the main strength of this study. A further strength is that swallowing and eating assessment was performed in 84% of the patients who fulfilled the inclusion criteria.

The sample size in this study was relatively small, which may have led to type II error. Furthermore, data regarding BMI, ASA score, delirium, habitual NMS, habitual swallowing difficulties, type of anesthesia, and CAS are not complete. Love et al. (2013) demonstrated an association between postoperative dysphagia and postoperative delirium [41] and Beric et al. (2019) that postoperative confusion predicted dysphagia post-surgery [39]. Meals et al. (2016) demonstrated that the ASA score was a meaningful predictor of dysphagia [42]. The fact that we could not observe any association between swallowing and eating difficulties and delirium and the ASA score, respectively, in our study may be explained by the small sample size and the missing data.

As mentioned earlier, patients operated for hip fracture are characterized by high age [26], and therefore cognitive impairment in this patient group is likely. In Denmark, screening for cognitive impairment during hospitalization is not common practice, and therefore screening for cognitive function was not done in this study, though it would have been relevant.

Due to the cross-sectional design in this study, no detection of causal relationships is possible. Furthermore, because there was no follow-up, possible changes in swallowing and eating difficulties over time, for instance as a result of physical training, were not identified.

Habitual NMS and habitual swallowing difficulties were obtained based on self-reporting from the patient, a relative, or a care assistant, and information bias is, therefore, possible. Simultaneously, an underestimation of the habitual swallowing difficulties is possible since patients were not tested before the operation.

Video fluoroscopy and fiberoptic endoscopic evaluation of swallowing are objective assessments of swallowing function [56]. It was not possible to use these assessments in our clinical setting. Instead, to examine swallowing difficulties, we used V-VST, which is translated into Danish but not yet validated in Denmark. V-VST was chosen because studies have shown a strong correlation between video fluoroscopy and V-VST [43] and given the fact that V-VST has been recommended in reviews [57,58]. V-VST uses a decrease of oxygen saturation ≥3% to detect silent aspiration, which is not a reliable indicator [59]. Furthermore, pharyngeal residue is impossible to visualize in a bedside screening but was in this study based on the question of patient experience.

To examine eating difficulties we used MEOF-II. MEOF-II is validated and recommended as a measurement for the performance of a meal [3,46]. A study recently published provides support for the reliability and validity of the Danish version of the MEOF II [47]. MEOF-II is not validated for detecting dysphagia, and the tool has no focus on the viscosity of the food, but the occupational therapist who performed the MEOF-II used food and fluids with different viscosities.

Patients not screened for swallowing and eating difficulties were more likely to live habitually in a nursing home and to be delirious postoperatively. As mentioned earlier, living in a residential aged care facility before hospital admission and presence of postoperative delirium has previously been found to be associated with postoperative dysphagia in elderly patients with hip fracture [39,41]. Patients not screened waited, on average, for a longer time from admission to surgery, and more of the patients not screened underwent surgery in general anesthesia. A previous study has shown that waiting time to surgery is correlated with an increased risk of serious adverse events during the hospital stay in patients with hip fracture [60], adverse events out of which some are previously documented to be associated with dysphagia [41]. Finally, patients not screened had a lower CAS day 1 after surgery. CAS is a valid tool for evaluating basic mobility in patients with hip fracture [61], and a lower CAS in patients not screened indicates a less independent and thereby more fragile group than patients screened. All these factors can lead to selection bias, underestimating the prevalence of swallowing and eating difficulties.

## 5. Conclusions

Swallowing and eating difficulties were highly prevalent (77%) in the elderly postoperative hip fracture population. Living in a nursing home before hospital admission, a low habitual NMS, and the absence of cardiac comorbidity were found to be significantly associated with swallowing and eating difficulties. The results indicate that systematic assessment of swallowing and eating difficulties in the elderly operated for hip fracture may be important to ensure effectivity and safety during meals and thereby improve the requisites of sufficient nutrition, prevent secondary complications, and improve the prognosis for the patients. However, because the study was conducted as a pilot study and thereby had a small sample size, the results needs to be tested in a larger study.

## Figures and Tables

**Table 1:** Baseline demographics and clinical characteristics and comparison of these factors of patients screened and patients not screened for swallowing and eating difficulties.

Population Variable	Patients Screened (*n* = 78)	Patients Not Screened (*n* = 15)	*p*-Value
Gender			0.484
Male	24 (30.8)	6 (40.0)	
Female	54 (69.2)	9 (60.0)	
Age (year), mean (SD)	81.4 (7.8)	82.7 (10.9)	0.592
Height (cm), mean (SD)	166.7 (10.6)	169.1 (10.4)	0.416
Weight (kg), mean (SD)	67.5 (14.9)	65.8 (12.6)	0.695
Body mass index, mean (SD)	24.3 (4.3)	23.0 (4.3)	0.320
Habitual housing form			0.048
Own residence	62 (79.5)	8 (53.3)	
Nursing home	16 (20.5)	7 (46.7)	
Comorbidity			
Neurological comorbidity	27 (34.6)	8 (53.3)	0.244
Respiratory comorbidity	19 (24.4)	4 (26.7)	1.000
Cardiac comorbidity	47 (60.3)	5 (33.3)	0.087
Ear, nose, or throat comorbidity	6 (7.7)	1 (6.7)	1.000
Other comorbidity	69 (88.5)	13 (86.7)	1.000
American Society of Anesthesiologists score			0.360
ASA I	2 (3.3)	1 (12.5)	
ASA II	27 (45.0)	3 (37.5)	
ASA III	29 (48.3)	4 (50.0)	
ASA IV	2 (3.3)	0 (0.0)	
Mean (SD)	2.5 (0.6)	2.4 (0.7)	0.557
Delirium			0.024
Yes	1 (5.0)	2 (28.6)	
No	19 (95.0)	5 (71.4)	
Habitual New Mobility Score			0.394
0	0 (0.0)	0 (0.0)	
1	1 (2.3)	0 (0.0)	
2	3 (6.8)	1 (33.3)	
3	5 (11.4)	0 (0.0)	
4	6 (13.6)	0 (0.0)	
5	3 (6.8)	0 (0.0)	
6	7 (15.9)	2 (66.7)	
7	5 (11.4)	0 (0.0)	
8	1 (2.3)	0 (0.0)	
9	13 (29.5)	0 (0.0)	
Mean (SD)	6.0 (2.5)	4.7 (2.3)	0.385
Habitual swallowing difficulties			0.112
Yes	3 (4.4)	1 (10.0)	
No	65 (95.6)	9 (90.0)	
Fracture type			0.157
Pertrochanteric	36 (46.2)	4 (26.7)	
Subtrochanteric	5 (6.4)	0 (0.0)	
Collum	37 (47.4)	11 (73.3)	
Time from admission to surgery (hours), mean (SD)	12.7 (9.8)	18.9 (10.8)	0.030
Type of anesthesia			0.006
General	36 (46.2)	9 (60.0)	
Spinal	40 (51.3)	3 (20.0)	
Other kind/unknown	2 (2.6)	3 (20.0)	
Surgery type			0.342
Arthroplasty	25 (32.1)	6 (40.0)	
Intramedullary nail	17 (21.8)	5 (33.3)	
Dynamic hip screw	29 (37.2)	2 (13.3)	
Splint	7 (9.0)	2 (13.3)	
Time from surgery to swallowing and eating assessment (hours), mean (SD)	30.4 (19.0)		
Cumulated Ambulation Score day 1 after surgery			0.003
0	1 (1.5)	2 (20.0)	
1	2 (2.9)	1 (10.0)	
2	23 (33.8)	2 (20.0)	
3	27 (39.7)	1 (10.0)	
4	5 (7.4)	2 (20.0)	
5	0 (0.0)	1 (10.0)	
6	10 (14.7)	1 (10.0)	
Mean (SD)	3.1 (1.4)	2.7 (2.1)	0.467

Data are presented as *n* (%) unless otherwise indicated.

**Table 2:** Comparison of baseline demographics and clinical characteristics of patients with and without swallowing and eating difficulties.

Population Variable	Swallowing and Eating Difficulties (*n* = 60)	No Swallowing and Eating Difficulties (*n* = 18)	*p*-Value
Gender			0.754
Male	19 (31.7)	5 (27.8)	
Female	41 (68.3)	13 (72.2)	
Age (year), mean (SD)	81.1 (8.2)	82.4 (6.5)	0.544
Height (cm), mean (SD)	166.2 (10.8)	168.3 (10.1)	0.483
Weight (kg), mean (SD)	66.7 (16.0)	69.8 (10.9)	0.442
Body mass index, mean (SD)	24.0 (4.4)	25.3 (4.0)	0.267
Habitual housing form			0.014
Own residence	44 (73.3)	18 (100.0)	
Nursing home	16 (26.7)	0 (0.0)	
Volume-Viscosity swallow test	38 (63.3)		
Impaired safety	17 (28.3)		
Impaired efficacy	32 (53.3)		
Minimal Eating Observation Form-II	48 (80.0)		
Ingestion	48 (80.0)		
Sitting position	38 (63.3)		
Manipulation of food on the plate	23 (38.3)		
Transport of food to the mouth	22 (36.7)		
Deglutition	38 (63.3)		
Manipulation of food in the mouth	22 (36.7)		
Swallowing	27 (45.0)		
Ability to chew	30 (50.0)		
Energy/appetite ^a^	19 (31.7)		
Comorbidity			
Neurological comorbidity	24 (40.0)	3 (16.7)	0.068
Respiratory comorbidity	16 (26.7)	3 (16.7)	0.386
Cardiac comorbidity	32 (53.3)	15 (83.3)	0.023
Ear, nose, or throat comorbidity	5 (8.3)	1 (5.6)	0.698
Other comorbidity	55 (91.7)	14 (77.8)	0.106
American Society of Anesthesiologists score			0.489
ASA I	1 (2.3)	1 (6.3)	
ASA II	18 (40.9)	9 (56.3)	
ASA III	23 (52.3)	6 (37.5)	
ASA IV	2 (4.5)	0 (0.0)	
Mean (SD)	2.6 (0.6)	2.3 (0.6)	0.128
Delirium			0.532
Yes	1 (5.9)	0 (0.0)	
No	16 (94.1)	3 (100.0)	
Habitual New Mobility Score			0.320
0	0 (0.0)	0 (0.0)	
1	1 (3.1)	0 (0.0)	
2	3 (9.4)	0 (0.0)	
3	5 (15.6)	0 (0.0)	
4	4 (12.5)	2 (16.7)	
5	2 (6.3)	1 (8.3)	
6	6 (18.8)	1 (8.3)	
7	4 (12.5)	1 (8.3)	
8	1 (3.1)	0 (0.0)	
9	6 (18.8)	7 (58.3)	
Mean (SD)	5.4 (2.5)	7.4 (2.1)	0.018
Habitual swallowing difficulties			0.595
Yes	3 (5.8)	0 (0.0)	
No	49 (94.2)	16 (100.0)	
Fracture type			0.964
Pertrochanteric	28 (46.7)	8 (44.4)	
Subtrochanteric	4 (6.7)	1 (5.6)	
Collum	28 (46.7)	9 (50.0)	
Time from admission to surgery (hours), mean (SD)	13.2 (9.8)	11.1 (9.7)	0.422
Type of anesthesia			0.520
General	29 (48.3)	7 (38.9)	
Spinal	29 (48.3)	11 (61.1)	
Other kind/unknown	2 (3.3)	0 (0.0)	
Surgery type			0.285
Arthroplasty	20 (33.3)	5 (27.8)	
Intramedullary nail	15 (25.0)	2 (11.1)	
Dynamic hip screw	19 (31.7)	10 (55.6)	
Splint	6 (10.0)	1 (5.6)	
Time from surgery to swallowing and	30.5 (19.5)	29.8 (22.4)	0.894
eating assessment (hours), mean (SD)			
Cumulated Ambulation Score day 1 after surgery			0.933
0	1 (1.9)	0 (0.0)	
1	2 (3.8)	0 (0.0)	
2	18 (34.6)	5 (31.3)	
3	20 (38.5)	7 (43.8)	
4	4 (7.7)	1 (6.3)	
5	0 (0.0)	0 (0.0)	
6	7 (13.5)	3 (18.8)	
Mean (SD)	3.0 (1.4)	3.3 (1.5)	0.445

Data are presented as n (%) unless otherwise indicated. ^a^ For MEOF-II energy/appetite, only the sub-question “energy to eat until having satisfied hunger” was included.
